# Intracranial epidural hematoma following lumbar puncture

**DOI:** 10.1007/s13760-021-01621-5

**Published:** 2021-04-11

**Authors:** Iryna Vynogradova, Steffen Ulrich Pauli, Josef Georg Heckmann

**Affiliations:** 1 Department of Neurology, Municipal Hospital Landshut, Robert-Koch Str. 1, 84034 Landshut, Germany; 2 Department of Neurosurgery, Municipal Hospital Landshut, 84034 Landshut, Germany; 3 Medical Faculty, University of Erlangen-Nuremberg, 91054 Erlangen, Germany

**Keywords:** Intracranial epidural hematoma, Lumbar puncture, Traumatic spinal tap

Despite technical advances and changes in the indication, lumbar puncture (LP) is an important and indispensable procedure for diagnostic and therapeutic purposes in neurological disorders. LP is generally considered safe, but complications such as post-puncture headache, hypoacusis, cranial nerve palsies and subdural hematoma can occur [1, 2]. Recently, the risk of spinal epidural hematoma (EDH) following LP was evaluated 0.2% among patients without coagulopathy and 0.23% among those with coagulopathy [3]. By our own clinical observation and one additional reported case in the literature, we wish to red-flag that even *intracranial* EDH can occur after LP [4].

A 32-year-old man was admitted due to a left-sided headache and neck pain with an intensity of 8 out of 10 on a numeric pain scale (10 is severest pain) without a fever. He denied a preceding trauma, and drug or alcohol abuse. Some days earlier, he contacted the emergency department due to a headache, which was judged as a headache due to arterial hypertension. His history was otherwise unremarkable. On admission, he reported pronounced neck pain and left-sided headache without focal neurological abnormalities. The blood pressure was 128/88 mm Hg. The routine laboratory tests including coagulation studies, platelet count, and his computed tomography (CT) brain scan were normal (Fig. 1a). To exclude meningitis and CT-negative subarachnoid hemorrhage LP was indicated. A LP with a 22-gauche atraumatic Sprotte needle (0.7 mm diameter) did not succeed to retrieve CSF. Therefore, the puncture was repeated with a 20-gauge Quincke needle (0.9 mm diameter) with success and showed normal results. Clinically, there were no signs of hemorrhage at this time. After analgesic medication, the patient reported some improvement that was then followed by a relapse. Cerebral magnetic resonance imaging (MRI) was performed the next day to exclude cerebral venous thrombosis, but demonstrated a marked frontal EDH (Fig. 1b), which was treated neurosurgically. Intraoperatively slight venous trickling on the surface of the dura mater was identified as the bleeding source. The patient recovered prompt and was ambulatory after a further 4 days.

**Fig. 1:**
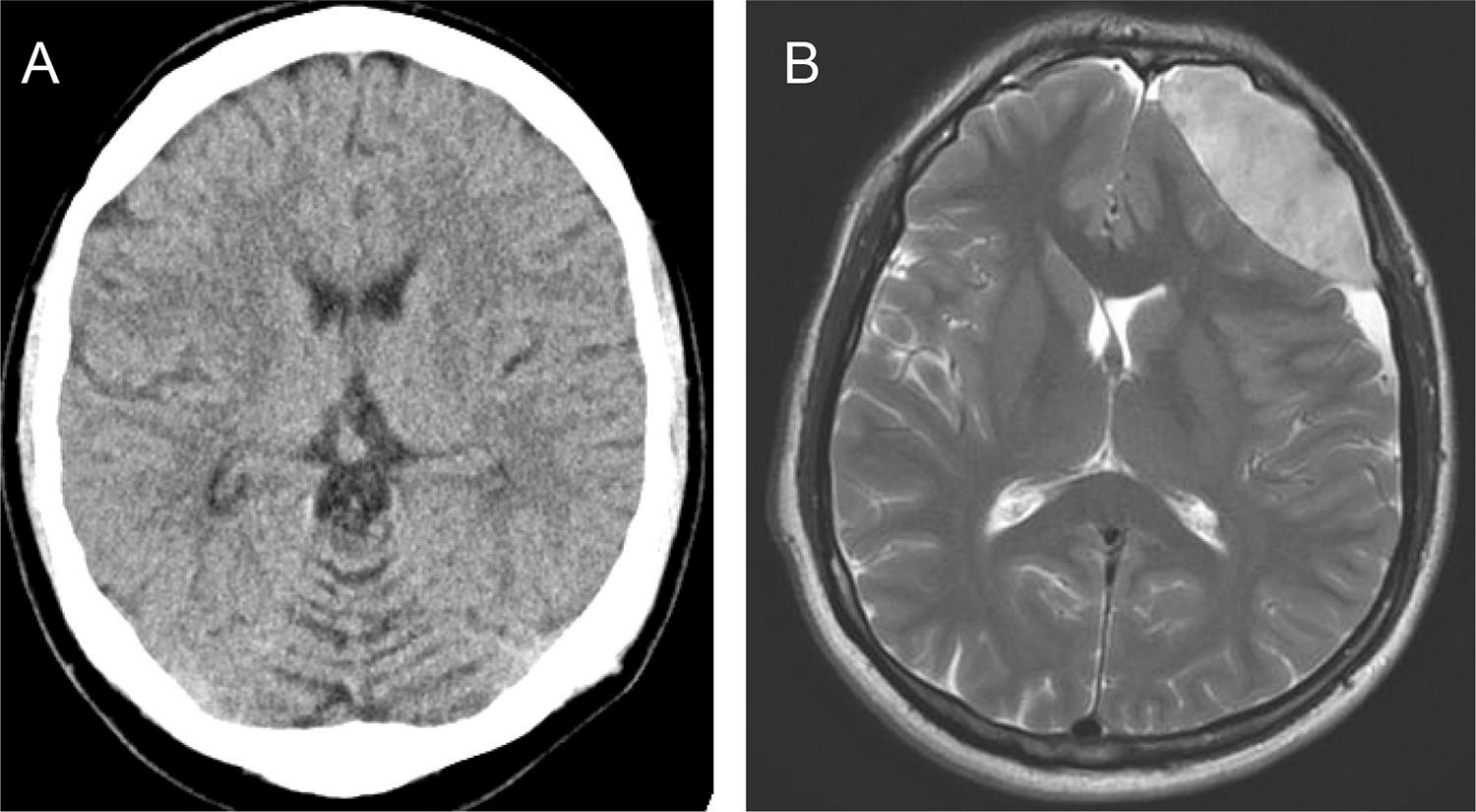
**a** Normal cranial computed tomography in a 32-year-old man on admission due to a headache and neck pain. **b** T_2_-weighted MRI revealing pronounced frontal epidural hematoma (7.5 × 2.5 × 5 cm) with consecutive midline shift of 10 mm following lumbar puncture. (Courtesy of Hans-Peter Dinkel, MD, PhD, Radiological Institute, Municipal Hospital Landshut)

Non-traumatic intracranial EDH is associated with infection, abscess, coagulopathy, hemorrhagic tumors, or vascular malformations, which could be excluded by our patient’s history and ancillary examinations [5]. In addition, a spontaneous EDH due to cerebral venous thrombosis seems improbable as the MRI was otherwise normal. Rather, we see a connection between the LP and the EDH. In a literature search, we identified one similar case [4]. Rapid shifts in CSF pressure following LP and consequent CSF hypotension are discussed as pathophysiological causes which lead to the dura detaching from the skull and inducing EDH [6]. Theoretically, a 20-gauge needle opening allows a flow of 36 ml CSF per minute, thus after removing the needle, a notable amount of CSF can leak from the dural sac, if the dura tissue does not close sufficiently. A similar pathophysiological moment of brain shift with loss of tamponade effect is discussed in delayed EDH, following contralateral epidural hematoma evacuation [7]. In our patient, we cannot absolutely exclude a LP-independent spontaneous EDH, but the course of disease with inconspicuous cranial CT at the time of LP speaks strongly against an EDH in nascendi. The frontal location and the younger age of the patient also indicate a relationship between LP and EDH as discussed by Patel et al. [4].

Taken together, spontaneous EDH is an extremely rare condition but should be considered as a possible complication of CSF loss during surgery or even after LP as in our case [4, 6, 8]. Furthermore, our observation suggests that LP should be performed under in-hospital conditions with a sufficient observation period to detect such complications quickly and react promptly. However, this should not lead to omit LP if indicated. In our own institution, with increasing number of diagnostic LP over a 30-year period (in 2019, 799 diagnostic lumbar punctures), such a condition has been observed for the first time.

## References

[1] Costerus JM, Brouwer MC, van de Beek D (2018). Technological advances and changing indications for lumbar puncture in neurological disorders. Lancet Neurol.

[2] Vos PE, de Boer WA, Wurzer JA, van Gijn J (1991). Subdural hematoma after lumbar puncture: two case reports and review of the literature. Clin Neurol Neurosurg.

[3] Bodilsen J, Mariager T, Vestergaard HH, Christiansen MH, Kunwald M, Lüttichau HR, Kristensen BT, Bjarkam CR, Nielsen H (2020). Association of lumbar puncture with spinal hematoma in patients with and without coagulopathy. JAMA.

[4] Patel BA, Williams NR, Pritchard PB (2013). Unique case of “post-lumbar puncture headache”. Headache.

[5] Papdopoulos MC, Dyer A, Hardwidge C (2001). Spontaneous extradural haematoma with sinusitis. J R Soc Med.

[6] Li ZJ, Sun P, Dou YH, Lan XL, Xu J, Zhang CY, Wang JP (2012). Bilateral supratentorial epidural hematomas: a rare complication in adolescent spine surgery. Neurol Med Chir (Tokyo).

[7] Gregori F, Santoro G, Mancarella C, Piccinilli M, Domenicucci M (2019). Development of a delayed acute epidural hematoma following contralateral epidural hematoma evacuation: case report and review of the literature. Acta Neurol Belg.

[8] Nemir J, Peterkovic V, Trninic I, Dornazet I, Baric H, Vukic M (2018). Intracranial epidural haematoma following surgical removal of a giant lumbosacral schwannoma: a case report and review of the literature. Pediatr Neurosurg.

